# The therapeutic potential of three-dimensional multipotent mesenchymal stromal cell spheroids

**DOI:** 10.1186/s13287-017-0558-6

**Published:** 2017-04-26

**Authors:** Yuriy Petrenko, Eva Syková, Šárka Kubinová

**Affiliations:** 1 Department of Biomaterials and Biophysical Methods, Institute of Experimental Medicine AS CR v. v. i, Vídeňská 1083, 14220 Prague 4-Krč, Czech Republic; 2 Department of Neuroscience, Charles University, Second Faculty of Medicine, V Uvalu 84, 15006 Prague, Czech Republic

**Keywords:** Multipotent mesenchymal stromal cells, Three-dimensional spheroids, Clinical-grade manufacturing, Paracrine activity, Survival, Therapeutic efficiency, Pre-clinical studies

## Abstract

The efficiency of clinical trials involving transplantation of multipotent mesenchymal stromal cells (MSCs) is often insufficient due to harsh conditions present within the target tissue including hypoxia, low nutrient supply as well as inflammatory reactions. This indicates the necessity for optimization of cell-based therapy approaches which might include either modification of the cell manufacturing process or specific cell pretreatment procedures prior to transplantation. Recent reports confirm evidence that the aggregation of MSCs into three-dimensional (3D) multicellular spheroids results in enhancement of the overall therapeutic potential of cells, by improving the anti-inflammatory and angiogenic properties, stemness and survival of MSCs after transplantation. Such an MSCs spheroid generation approach may open new opportunities for the enlargement of MSCs applications in clinical research and therapy. However, the unification and optimization of 3D spheroid generation techniques, including the selection of appropriate clinical-grade culture conditions and methods for their large-scale production, are still of great importance. The current review addresses questions regarding therapeutic-associated properties of 3D multicellular MSCs spheroids in vitro and during preclinical animal studies, with special attention to the possibilities of translating these research achievements toward further clinical manufacturing and applications.

## Background

Multipotent mesenchymal stromal cells (MSCs) represent unique opportunities in cellular therapy due to their ability to stimulate the regeneration of damaged tissues and organs. MSCs are a fibroblast-like population of progenitor cells, characterized by specific immunophenotype (CD105^+^, CD73^+^, CD90^+^, CD34^–^, CD45^–^, HLA-DR^–^, CD14^–^) and multilineage differentiation potential [1]. While the positive mechanism of MSC therapy was initially associated with their differentiation into target cell types after transplantation, recent clinical studies have mainly focused on the ability of MSCs to act as an effective biological stimulant with high paracrine activity. As currently considered, the plasticity of therapeutic action of MSCs is associated with their ability to secrete molecules in response to microenvironmental and hormonal signals. The secretome of MSCs comprises a number of growth factors and cytokines, as well as micro-vesicles and exosomes, which are involved in the transfer of proteins and miRNA to other cells [2]. The ability to modify the extracellular matrix within the tissue or to transfer mitochondria through cell–cell interactions also confirms the therapeutic potency of MSCs. Such interaction between MSCs and surrounding cells and tissues can promote the formation of new blood vessels (angiogenic effect), prevent cell death (anti-apoptotic) and modulate inflammation and immune responses, providing a microenvironment necessary for normal development and differentiation of resident stem cells [3].

However, the beneficial effects of MSC-based therapies in small-scale clinical studies are often not substantiated by large randomized controlled clinical trials [4]. The survival and engraftment of transplanted MSCs is usually inadequate, due to hypoxia, low nutrient supply or inflammatory reactions within the target tissues. Moreover, the excessive expansion needed to obtain therapeutically relevant cell numbers can be associated with a decrease in the immunomodulatory potential of MSCs, an increase in senescence and thus lower survival after transplantation, compared with minimally expanded cells. Such evidence strongly indicates the need for further optimization of cell-based therapy approaches, by modifying the cell manufacturing process or by inclusion of specific cell pretreatment procedures prior to transplantation. Recently, an alternative and comparably easy method for enhancing the therapeutic potential of MSCs has been demonstrated, which is based on the preparation and application of cells as multicellular spheroids [5]. Starting from early 1940, spheroid cultures have been used successfully for embryonic or tumor cells in attempts to understand the morpho/organogenesis of malignancy, and the effects of different experimental therapeutics on tumor cells and three-dimensional (3D) organoids [6]. Subsequent attempts significantly broadened the directions for 3D spheroid culture application, including 3D tissue modeling for drug discovery, toxicology studies and regenerative medicine applications. Besides “classical” spheroid cultures involving cancer cell lines, a large variety of noncancer cell types have been employed for 3D spheroid generation, predominantly of hepatic, cardiac and neural tissue phenotype.

In this review, we discuss the reported data concerning the therapeutic-associated properties of 3D multicellular MSC spheroids in vitro and after experimental transplantation, with special attention to the possibilities of translating these research achievements toward further clinical manufacturing and applications.

### Enhanced therapeutic potential of spheroid MSCs in vitro

**Fig. 1:**
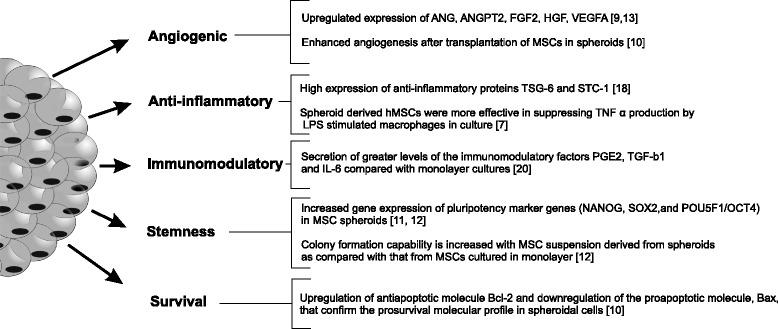
Therapeutic-associated changes in properties of MSCs after aggregation into 3D spheroids. *ANG* angiogenin, *ANGPT-2* angiopoietin-2, *FGF* fibroblast growth factor, *HGF* hepatocyte growth factor, *VEGF* vascular endothelial growth factor, *MSC* multipotent mesenchymal stromal cell, *TSG-6* tumor necrosis factor-inducible gene 6 protein, *STC-1* stanniocalcin-1, *TNFα* tumor necrosis factor alpha, *PGE-2* prostaglandin E2, *TGF* transforming growth factor, *IL* interleukin, *Sox2* sex determining region Y-box 2, *Oct4* octamer-binding transcription factor 4, *BAX* Bcl-2-associated X protein, *BCL-2* B-cell lymphoma 2

The 3D cell organization provides enhanced cell–cell interactions and closely mimics the natural microenvironment of a tissue, compared with traditional 2D monolayer cultures. Short-term culture of MSCs in a 3D environment was shown to have a nonsignificant effect on the level of MSC-specific immunophenotypic marker expression. The changes demonstrated in several studies [7, 8] were mainly associated with differences in cellular adhesion between the usual 2D tissue culture substrate and 3D spheroid cultures. At the same time, such an approach was confirmed to enhance anti-inflammatory and angiogenic properties of MSCs [9, 10], increase stemness [11, 12] and facilitate differentiation into different cell lineages, as well as improve the survival of cells after transplantation [10] (Fig. 1).

The expression of angiogenic growth factors and cytokines, such as angiogenin (ANG), fibroblast growth factor 2 (FGF-2), angiopoietin 2 (ANGPT-2), VEGF and hepatocyte growth factor (HGF), are significantly increased in MSC spheroid cultures [9, 13]. Enhanced proangiogenic properties of MSC spheroids have been confirmed in different animal models, involving either cell transplantation or implantation of MSC spheroid-containing tissue engineered structures. Murphy et al. [14] reported that MSC spheroids within the fibrin gel secrete 100-times higher levels of VEGF, compared with the same number of dissociated cells. 3D spheroid formation of MSCs was found to upregulate E-cadherin expression, which was responsible for enhanced VEGF secretion via the ERK/AKT signaling pathway [15]. The generation of 3D multicellular MSC spheroids has been shown to be associated with increased expression of CXCR4, positively affecting the adhesion of spheroid-derived MSCs to endothelial cells [9], which may represent one of the major events during cell homing after infusion. Similar observations have been shown by Cheng et al. [11], where two-times higher expression of CXCR4 receptor was obtained for spheroid-derived adipose tissue MSCs compared with conventionally cultured cells.

Previously, it was demonstrated that intravenously infused MSCs are trapped in the lungs as micro emboli, with subsequent activation of anti-inflammatory TNFα-stimulated gene/protein 6 (TSG-6) secretion after 12–24 hours of entrapment [16]. In mouse models, such TSG-6 secretion decreased the inflammatory reactions in the heart. However, MSCs in initial suspension did not produce TSG-6, which initiated studies regarding the modification of MSCs properties to obtain cells with a high anti-inflammatory potential. It has been shown that the spheroid culture of MSCs leads to significant upregulation of TSG-6 and stanniocalcin-1 (STC-1) expression, providing the possibility for comparably simple activation of MSCs toward the production of anti-inflammatory proteins [7, 17]. The inflammatory environment, such as the presence of interferon gamma (IFN-γ), tumor necrosis factor alpha (TNFα) or IL-1β, regulates MSCs immunomodulatory activity. It has been determined that 3D aggregation of MSCs activates prostaglandin E2 (PGE-2) and TSG-6 factor secretion, known to suppress macrophage inflammatory cytokine production [7, 17, 18]. Spheroid MSCs have been shown to secrete significantly greater levels of TGF-β1 and IL-6 compared with monolayer cultures, confirming enhanced immunomodulatory potential [19]. However, the expression of indoleamine-pyrrole 2,3-dioxygenase (IDO), one of the key factors involved in immunosuppression, is not significantly different between 2D and 3D spheroid cultures of MSCs [19]. Only after double treatment of spheroids with both IFN-γ and TNF-α a significant difference in IDO activity of spheroid cells was observed, compared with monolayer cultures. However, such an effect was strongly dependent on the culture medium used. Thus, further studies are needed to improve IDO expression within the 3D spheroid-based environment.

In addition to improved angiogenic and anti-inflammatory/immunomodulatory properties, aggregation of MSCs into 3D spheroids provides a significant impact on the stemness characteristics of cells. Spheroid-derived adipose tissue MSCs showed a significantly higher expression of *Nanog*, *Sox2* and *Oct4* genes, compared with monolayer cultures [11, 12]. Moreover, MSCs derived from spheroids have been shown to have higher expansion and colony-forming activity [11]. Guo et al. [12] showed that a 3D environment promoted the expression of miRNA involved in multipotency of MSCs. Spheroid-derived MSCs showed enhanced ability for differentiation toward neural and hepatic cells [20, 21]. Yeh et al. [22] showed the enhancement of WNT signaling-related gene expression in spheroid-derived MSCs.

The poor survival of transplanted cells is one of the major problems that occurred during cell therapy trials. The hypoxic conditions present in the injured region may cause irreversible ischemic injury to MSCs after transplantation. It has been reported by Bhang et al. [10] that MSCs spheroids better survive the ischemic conditions, compared with 2D expanded cells. It has been reported that adipose tissue MSCs after 3D spheroid culture express more levels of hypoxia-inducible factor 1 and manganese superoxide dismutase, which correlated with improved resistance to oxidative stress-induced apoptosis [23]. Besides improved tolerance to hypoxia, significant upregulation of anti-apoptotic molecule BCL-2, and downregulation of proapoptotic molecule Bax, has been shown for spheroid-derived MSCs, confirming the prosurvival properties of these cells [5, 10]. Additionally, several studies demonstrated that spheroid MSCs, independent of the tissue of origin, are characterized by a significant size decrease (to about 0.25–0.5 of the volume of an average monolayer cultured cell); fewer cells are thus entrapped in the lungs after intravenous injection, when compared with conventionally derived MSCs [7, 24].

The possible mechanisms of functional improvement of MSCs within 3D spheroids have been thoroughly reviewed recently [25, 26]. Briefly, the organization of cells into 3D multicellular spheroids results in a variety of changes in the cellular microenvironment, compared with conventional 2D culture. The changes in cell–matrix and cell–cell interactions lead to significant rearrangement of physical forces acting on each cell within the 3D spheroid, changing the polarization, cytoskeleton organization and morphology of cells. Substrate stiffness and elasticity significantly influence paracrine properties of MSCs [26, 27]. The differences in strain and rigidity that occurred during spheroid formation compared with standard tissue culture plastic were shown to be significant [26, 28]. At the same time, Abdeen et al. [29] showed that the variation in substrate stiffness resulted in changes of proangiogenic signaling of MSCs. Cell shape also determines the switch in MSC lineage commitment, which was shown to be mediated through the RhoA-ROCK signaling pathway [30]. Increased cell–cell contact within spheroids upregulates the expression of cadherins (E-cadherin, N-cadherin, cadherin 11) and gap junction proteins (Connexin-43), which were confirmed to define the lineage specificity of MSCs [31]. Lee et al. [15] showed that E-cadherin is a key mediator in the formation of human umbilical cord blood MSC spheroids, which regulates the proliferative and paracrine activity of cells through the ERK/AKT signaling pathway. Mild hypoxia within the inner mass of 3D spheroids has been suggested to act as an initiator of prosurvival and angiogenic factor expression [32]. However, in a recent study Murphy et al. [33] showed that the oxygen tension values varied less than 10% from the outer diameter of spheroids 353 ± 18 μm in size, providing evidence that the enhanced function of MSCs within 3D spheroids is not oxygen mediated. Enhanced ECM secretion within the 3D spheroid provides a favorable environment for local growth factor and cytokine enrichment, supporting autocrine signaling. Increased autophagy protects cells from environmental stresses, and thus may improve survival and prevent early senescence of MSCs [34]. All of these mentioned factors could play important roles in the regulation of MSC differentiation and paracrine activity; however, the exact mechanisms that drive such significant changes in MSC properties during 3D spheroidal culture remain to be defined.

### Therapeutic properties of MSC spheroids in preclinical animal studies

**Fig. 2:**
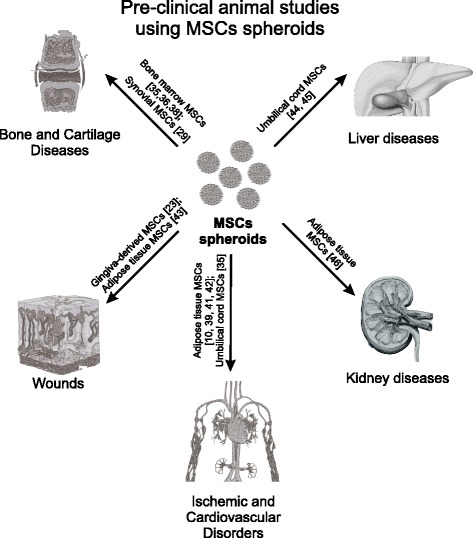
Preclinical animal studies using MSCs spheroids. *MSCs* multipotent mesenchymal stromal cell

These recently reported properties of spheroid MSCs initiated many preclinical studies, which involved a variety of animal models, mainly directed at osteochondral diseases, ischemic and cardiovascular disorders and wound healing (Fig. 2). After a subcutaneous injection of MSC aggregates into nude mice, significantly larger and denser ectopic bone formation was observed compared with dissociated cell suspension [35, 36]. Upregulation of chondrogenesis-related, anti-apoptotic and anti-inflammatory genes was observed in aggregated MSCs, which provided successful cartilage regeneration after transplantation into a knee joint [37]. Suenaga et al. [38] showed the application of bone marrow MSC spheroids for the repair of calvarial defects in nude rats. Micro-computed tomography and immunohistochemical staining for osteocalcin/osteopontin indicated the formation of new, full-thickness bones at the implantation sites in the MSCs spheroid group [38].

Following intramuscular transplantation to ischemic hind limbs of athymic mice, 3D spheroids of adipose tissue MSCs improved cell survival, angiogenic factor secretion, neovascularization and limb survival compared with MSCs dissociated suspensions [10]. As a result, the transplantation of MSCs spheroids significantly increased the number of microvessels and smooth muscle α-actin-positive vessels in the ischemic limbs of mice, and attenuated limb loss and necrosis [10]. Lee et al. [39] studied the expression of the proliferation marker “proliferating cell nuclear antigen” (PCNA) in ischemic tissues transplanted with monolayer MSCs or MSC spheroids. Expression of PCNA was higher in ischemic tissues transplanted with MSC spheroids than in tissues transplanted with monolayer MSCs.

Enhanced engraftment after transplantation of 3D umbilical cord blood MSC aggregates was observed in the rat myocardial infarction model [15]. The authors observed endothelial and cardiomyocyte differentiation of DiI-labeled MSCs after transplantation. The MSCs spheroid group showed a superior heart function improvement in a rat model after intramyocardial injection into the peri-infarct areas, compared with MSC suspensions [40]. The intramyocardial transplantation of human adipose tissue MSC spheroids in the myocardial infarction porcine model improved the heart function in 88.8% of animals [41]. The authors revealed no arrhythmogenic, embolic or neurological events, but detected a myocardial retention of cells, confirmed by PCR and immunohistochemistry. Liu et al. [42] showed that rat adipose tissue MSC spheroids had 20-times higher levels of cardiac marker gene expression (*Gata4, Nkx2-5, Myh6 and Tnnt2*), compared with MSCs grown in monolayer, and provided better functional recovery in the myocardial infarction animal model 12 weeks post implantation.

Amos et al. [43] employed adipose tissue MSC spheroids for the treatment of full-thickness dermal wounds in leptin receptor-deficient mice. The significant increased rate of wound closure in diabetic mice was observed in 3D MSC spheroid groups, compared with an equal number of suspended MSCs. The authors showed that spheroid MSCs produced significantly higher amounts of ECM proteins: tenascin C, collagen VI alpha 3 and fibronectin [43]. Using an in-vivo murine model of chemotherapy-induced oral mucositis, Zhang et al. [23] showed that human gingiva-derived MSCs following spheroid generation provide better therapeutic efficacy compared with conventional cultures, reversing body weight loss and promoting regeneration of the disrupted epithelial lining of the mucositic tongues.

Several studies show the potential of spheroid MSCs application in hepatic regeneration and kidney injury models. The 3D cultured umbilical cord MSCs promoted secretion of IFN-γ and IL-6 but inhibited that of TNFα in the CCl_4_-induced acute liver failure mouse model, providing improved liver regeneration [44]. The increased potential of umbilical cord MSC spheroids for differentiation into the hepatic lineage has also been demonstrated by Talaei-Khozani et al. [45]. Xu et al. [46] applied 3D spheroids of human adipose tissue MSCs for acute kidney injury. When injected into the kidneys of model rats with ischemia–reperfusion-induced kidney injury, 3D spheroids were more beneficial in protecting the kidneys against cell apoptosis, reducing tissue damage, promoting vascularization and ameliorating renal function, compared with 2D cultured cells.

### Toward clinical-grade large-scale manufacturing of MSC spheroids

The presented properties of MSC spheroids and spheroid-derived cells in vitro and in vivo confirm a high potential for such approaches to be applied in regenerative medicine and tissue engineering. However, translation of laboratory and preclinical research into human trials needs adaptation and standardization of methods for cellular product manufacturing and application. Currently, most studies on the evaluation of MSC properties in 3D spheroid cultures employ fetal bovine serum (FBS) as a classical culture media supplement. However, the utilization of animal-derived products during the manufacturing of cellular therapeutics for human use is not recommended [47]. Only a few studies have so far been published on investigation into the effect of FBS substitutes of human origin or chemically defined medium on the MSC spheroid generation and functional properties of spheroid-derived cells. Recently, Yloslato et al. [17] showed that cell activation in a 3D environment depends critically on the culture medium. Spheroids can also be formed in chemically defined xeno-free medium, but the composition of the medium is critical for assembly of the cells into compact spheres and the patterns of genes expressed by MSCs. The authors showed that among commercially available chemically defined media only MesenCult™-XF promoted the formation of 3D spheroids in the absence of additional supplements. For other types of media, the inclusion of human serum albumin (HSA) appeared to be essential for nonforced formation of compact spheres with high-viability MSCs, and for the expression and secretion of potentially therapeutic anti-inflammatory (TSG-6, PGE2) and anti-cancer (IL-24) molecules [17]. Since 2005, the attention of researchers has gradually shifted toward the application of hPL for clinical-grade MSC manufacturing [48]. The supportive action of hPL-based products toward MSCs is explained by the complex effect of growth factors, mainly PDGF, bFGF and TGF-β; however, the presence of these factors was shown in only one out of three lots of FBS [49]. This confirms that there might be differences in MSCs properties related to changes in ex-vivo conditions. While the safety and preservation of MSCs identity is confirmed for both hPL-based and defined media, not enough information is available about the effect of xeno-free conditioning on the MSCs paracrine secretion profile in 2D and 3D environments. Recently, Zimmermann and McDevitt [19] showed that in-vitro culture of 3D MSC spheroids in FBS or chemically defined xeno-free media (MesenCult™-XF) resulted in a significantly different cell response to proinflammatory cytokines treatment. PGE2 and IDO secretion was found to be greater in spheroids cultured in FBS medium, compared with spheroids cultured in MesenCult-XF. The authors showed that human MSC spheroids in FBS-supplemented cultures showed low or no proliferation activity with increased paracrine secretion, while MSCs in xeno-free cultures have been characterized by significant cell growth but low paracrine secretion [19]. Therefore, the question about the optimization of xeno-free 3D culture conditions for the preparation of MSCs products with enhanced therapeutic properties is still highly relevant.

**Fig. 3:**
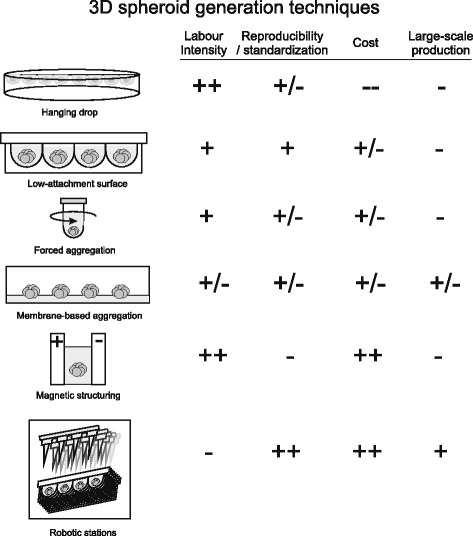
3D spheroid generation techniques

Besides adaptation of the cell culture environment and media composition, the widespread application of spheroid-derived MSCs requires development of efficient large-scale spheroid fabrication methods. Various spheroid generation techniques have already been established (Fig. 3). Among these, the most widespread are the hanging drop approach [7, 37, 50], application of low-adhesive substrates [39], membrane-based aggregation [11, 22] and the forced aggregation method [19].

Hanging drop cultures are a simple tool for the generation of cell spheroids, with a controlled cell number and spheroid size. The limitation of such a method for large-scale production of cellular spheroids is based on the limited area of standard culture dishes and labor-intensive processes of establishment, and the harvesting of generated 3D cultures.

The application of cell culture using low-attachment surfaces is another easy-to-use approach for spheroid generation; however, it leads to high variations in size and morphology of the produced cell spheroids. Forced aggregation techniques are widely used as a tool for generation of MSC aggregates for their further chondrogenic differentiation. However, the large-scale production of MSC spheroids using these techniques is economically ineffective and labor intensive.

Several biomaterial-assisted methods have been shown to promote spheroid generation, and polycationic chitosan membranes application is probably the most widely reported technique. Chitosan is a positively charged natural biocompatible polysaccharide, and the degree of its deacetylation will determine the cell adhesion properties and further spheroid-generating potential [51]. Yeh et al. [22] showed significantly higher expression levels of genes including *LIF*, *IL24*, *TP53*, *TGF-β3*, *PDGFRA* and *PTGS2* for MSCs grown on chitosan membranes, compared with nonadherent polyvinyl alcohol substrate. The authors proposed that enhanced gene expression may be attributed to the greater cell–substrate interaction for MSCs on chitosan films, through the upregulation of the calcium-associated genes [52]. Hsu and Huang [53] showed different WNT (Wnt3a, Wnt5a and Wnt5b) signaling when MSCs were cultured on pure chitosan films, or after surface modification with a different concentration of hyaluronan. The authors suggested that the variation of the substrate environment might predefine the differentiation properties of MSC spheroids through Wnt signaling. Recently, Yang et al. [34] reported the enhanced autophagy of MSCs within spheroids generated on chitosan surfaces. Such a feature was better expressed in chitosan-based spheroids compared with those obtained using low-attachment plates. The authors proposed that such enhanced MSC autophagy within 3D spheroids may determine their higher survival and therapeutic potential after transplantation.

Biomaterial-assisted spheroid generation is not limited by chitosan-based approaches. Messina et al. [54] reported the generation of cell spheroids using agarose and polycaprolactone membranes. The authors showed that spheroids underwent faster fusion and maturation on polycaprolactone membrane, compared with agarose. Wang et al. [55] showed the application of micropatterned substrates coated with photosensitized poly(ethylene glycol). Such cultured conditions provided an improved MSC differentiation potential. Zhang et al. [56] employed poly(l-glutamic acid)/chitosan scaffolds for in-situ generation of MSC spheroids for application in cartilage regeneration.

Another promising approach for spheroid generation is the application of magnetic structuring. Recently, Lewis et al. [57] showed that the use of superparamagnetic nanoparticles achieved magnetic levitation of MSCs within collagen 1 gels. As a result of 3 hours of incubation under external magnetic field, MSCs formed spheroids with a mean diameter of 259 μm, preserving their multipotent properties [57].

Several reports have shown the adaptation and robotization of common spheroid generation techniques for the large-scale high-throughput production of spheroids for drug discovery applications. Neto et al. [58] presented the development of the novel hanging drop system, based on the use of biomimetic super-hydrophobic flat substrates. By facing down the platform, it was possible to generate independent spheroid bodies in a high-throughput manner. Tung et al. [59] showed a high-throughput 3D spheroid culture within a 384 hanging drops array. Zhao et al. [60] used patterned nonadhesive poly(2-hydroxyethyl methacrylate) hydrogel films to guide the self-assembly of cells for high-throughput generation of multicellular spheroids. Application of such high-throughput approaches represents unique opportunities in drug discovery applications and toxicology studies; however, clinical use of 3D MSC spheroids will probably require significantly higher cell (or 3D spheroid) numbers, and thus further research is needed for the development of highly reproducible, simple and cost-effective techniques for large-scale production of these novel unique cellular therapeutics.

## Conclusions

Generation of 3D spheroids represents unique opportunities in the improvement of the therapeutic potential of MSCs derived from different human adult tissues. Enhanced stemness, anti-inflammatory and immunomodulatory properties as well as increased survival and anti-apoptotic features of MSCs in a 3D environment open new possibilities toward the enlargement of MSC applications in clinical research and therapy. However, unification and optimization of 3D spheroid generation techniques, including the selection of appropriate clinical-grade culture conditions and methods for their large-scale production, is still needed to provide highly efficient and reproducible MSCs-based therapeutics.

## Abbreviations

ANG: Angiogenin
ANGPT-2: Angiopoietin-2
BAX: Bcl-2-associated X protein
BCL-2: B-cell lymphoma 2
CXCR4: C-X-C chemokine receptor type 4
ERK/AKT: Extracellular signal-regulated kinase/protein kinase B
FBS: Fetal bovine serum
FGF: Fibroblast growth factor
HGF: Hepatocyte growth factor
hPL: Human platelet lysate
HSA: Human serum albumin
IDO: Indoleamine-pyrrole 2,3-dioxygenase
IL: Interleukin
MSC: Multipotent mesenchymal stromal cell
Oct4: Octamer-binding transcription factor 4
PCNA: Proliferating cell nuclear antigen
PDGF: Platelet-derived growth factor
PGE-2: Prostaglandin E2
Sox2: Sex determining region Y-box 2
STC-1: Stanniocalcin-1
TGF: Transforming growth factor
TNFα: Tumor necrosis factor alpha
TP53: Tumor protein 53
TSG-6: Tumor necrosis factor-inducible gene 6 protein
VEGF: Vascular endothelial growth factor
WNT: Wingless-type MMTV integration site family member

## References

[1] Dominici M, Le Blanc K, Mueller I, Slaper-Cortenbach I, Marini F, Krause D (2006). Minimal criteria for defining multipotent mesenchymal stromal cells. The International Society for Cellular Therapy position statement. Cytotherapy..

[2] Teixeira FG, Carvalho MM, Sousa N, Salgado AJ (2013). Mesenchymal stem cells secretome: a new paradigm for central nervous system regeneration?. Cell Mol Life Sci..

[3] Marquez-Curtis LA, Janowska-Wieczorek A, McGann LE, Elliott JAW (2015). Mesenchymal stromal cells derived from various tissues: biological, clinical and cryopreservation aspects. Cryobiology..

[4] English K, French A, Wood KJ (2010). Mesenchymal stromal cells: facilitators of successful transplantation?. Cell Stem Cell..

[5] Cesarz Z, Tamama K (2016). Spheroid culture of mesenchymal stem cells. Stem Cells Int..

[6] Mueller-Klieser W (1997). Three-dimensional cell cultures: from molecular mechanisms to clinical applications. Am J Physiol.

[7] Bartosh TJ, Ylöstalo JH, Mohammadipoor A, Bazhanov N, Coble K, Claypool K (2010). Aggregation of human mesenchymal stromal cells (MSCs) into 3D spheroids enhances their antiinflammatory properties. Proc Natl Acad Sci U S A..

[8] Park IS, Rhie JW, Kim SH (2014). A novel three-dimensional adipose-derived stem cell cluster for vascular regeneration in ischemic tissue. Cytotherapy..

[9] Potapova IA, Brink PR, Cohen IS, Doronin SV (2008). Culturing of human mesenchymal stem cells as three-dimensional aggregates induces functional expression of CXCR4 that regulates adhesion to endothelial cells. J Biol Chem..

[10] Bhang SH, Lee S, Shin J, Lee T, Kim B (2012). Transplantation of cord blood mesenchymal stem cells as spheroids enhances vascularization. Tissue Eng Part A..

[11] Cheng N-C, Wang S, Young T-H (2012). The influence of spheroid formation of human adipose-derived stem cells on chitosan films on stemness and differentiation capabilities. Biomaterials..

[12] Guo L, Zhou Y, Wang S, Wu Y (2014). Epigenetic changes of mesenchymal stem cells in three-dimensional (3D) spheroids. J Cell Mol Med..

[13] Potapova IA, Gaudette GR, Brink PR, Robinson RB, Rosen MR, Cohen IS, Doronin SV (2007). Mesenchymal stem cells support migration, extracellular matrix invasion, proliferation, and survival of endothelial cells in vitro. Stem Cells..

[14] Murphy KC, Fang SY, Leach JK (2014). Human mesenchymal stem cell spheroids in fibrin hydrogels exhibit improved cell survival and potential for bone healing. Cell Tissue Res..

[15] Lee EJ, Park SJ, Kang SK, Kim G-H, Kang H-J, Lee S-W (2012). Spherical bullet formation via E-cadherin promotes therapeutic potency of mesenchymal stem cells derived from human umbilical cord blood for myocardial infarction. Mol Ther..

[16] Lee RH, Pulin AA, Seo MJ, Kota DJ, Ylostalo J, Larson BL (2009). Intravenous hMSCs improve myocardial infarction in mice because cells embolized in lung are activated to secrete the anti-inflammatory protein TSG-6. Cell Stem Cell..

[17] Ylostalo JH, Bartosh TJ, Tiblow A, Prockop DJ (2014). Unique characteristics of human mesenchymal stromal/progenitor cells pre-activated in 3-dimensional cultures under different conditions. Cytotherapy..

[18] Bartosh TJ, Ylöstalo JH, Bazhanov N, Kuhlman J, Prockop DJ (2013). Dynamic compaction of human mesenchymal stem/precursor cells into spheres self-activates caspase-dependent IL1 signaling to enhance secretion of modulators of inflammation and immunity (PGE2, TSG6, and STC1). Stem Cells..

[19] Zimmermann JA, McDevitt TC (2014). Pre-conditioning mesenchymal stromal cell spheroids for immunomodulatory paracrine factor secretion. Cytotherapy..

[20] Hsueh Y-Y, Chiang Y-L, Wu C-C, Lin S-C (2012). Spheroid formation and neural induction in human adipose-derived stem cells on a chitosan-coated surface. Cells Tissues Organs..

[21] Cipriano M, Freyer N, Knöspel F, Oliveira NG, Barcia R, Cruz PE (2016). Self-assembled 3D spheroids and hollow-fibre bioreactors improve MSC-derived hepatocyte-like cell maturation in vitro. Arch Toxicol.

[22] Yeh H-Y, Liu B-H, Sieber M, Hsu S (2014). Substrate-dependent gene regulation of self-assembled human MSC spheroids on chitosan membranes. BMC Genomics.

[23] Zhang Q, Nguyen AL, Shi S, Hill C, Wilder-Smith P, Krasieva TB, Le AD (2012). Three-dimensional spheroid culture of human gingiva-derived mesenchymal stem cells enhances mitigation of chemotherapy-induced oral mucositis. Stem Cells Dev..

[24] Tsai A-C, Liu Y, Yuan X, Ma T (2015). Compaction, fusion, and functional activation of three-dimensional human mesenchymal stem cell aggregate. Tissue Eng Part A..

[25] Sart S, Tsai A-C, Li Y, Ma T (2014). Three-dimensional aggregates of mesenchymal stem cells: cellular mechanisms, biological properties, and applications. Tissue Eng Part B Rev..

[26] Follin B, Juhl M, Cohen S, Pedersen AE, Kastrup J, Ekblond A (2016). Increased paracrine immunomodulatory potential of mesenchymal stromal cells in 3D culture. Tissue Eng Part B Rev..

[27] Kusuma GD, Carthew J, Lim R, Frith JE (2017). Effect of the microenvironment on mesenchymal stem cell paracrine signaling: opportunities to engineer the therapeutic effect. Stem Cells Dev.

[28] Baraniak PR, Cooke MT, Saeed R, Kinney MA, Fridley KM, McDevitt TC (2012). Stiffening of human mesenchymal stem cell spheroid microenvironments induced by incorporation of gelatin microparticles. J Mech Behav Biomed Mater..

[29] Abdeen AA, Weiss JB, Lee J, Kilian KA (2014). Matrix composition and mechanics direct proangiogenic signaling from mesenchymal stem cells. Tissue Eng Part A..

[30] McBeath R, Pirone DM, Nelson CM, Bhadriraju K, Chen CS (2004). Cell shape, cytoskeletal tension, and RhoA regulate stem cell lineage commitment. Dev Cell..

[31] Shin CS, Lecanda F, Sheikh S, Weitzmann L, Cheng SL, Civitelli R (2000). Relative abundance of different cadherins defines differentiation of mesenchymal precursors into osteogenic, myogenic, or adipogenic pathways. J Cell Biochem..

[32] Bhang SH, Cho S-W, La W-G, Lee T-J, Yang HS, Sun A-Y (2011). Angiogenesis in ischemic tissue produced by spheroid grafting of human adipose-derived stromal cells. Biomaterials..

[33] Murphy KC, Hung BP, Browne-Bourne S, Zhou D, Yeung J, Genetos DC, Leach JK. Measurement of oxygen tension within mesenchymal stem cell spheroids. J R Soc Interface. 2017;14. doi:10.1098/rsif.2016.0851. 10.1098/rsif.2016.0851 PMC5332570 28179546

[34] Yang C-M, Huang Y-J, Hsu S-H (2015). Enhanced autophagy of adipose-derived stem cells grown on chitosan substrates. Biores Open Access..

[35] Ma D, Ren L, Liu Y, Chen F, Zhang J, Xue Z, Mao T (2010). Engineering scaffold-free bone tissue using bone marrow stromal cell sheets. J Orthop Res..

[36] Ma D, Zhong C, Yao H, Liu Y, Chen F, Li J (2011). Engineering injectable bone using bone marrow stromal cell aggregates. Stem Cells Dev..

[37] Suzuki S, Muneta T, Tsuji K, Ichinose S, Makino H, Umezawa A, Sekiya I (2012). Properties and usefulness of aggregates of synovial mesenchymal stem cells as a source for cartilage regeneration. Arthritis Res Ther..

[38] Suenaga H, Furukawa KS, Suzuki Y, Takato T (2015). Bone regeneration in calvarial defects in a rat model by implantation of human bone marrow-derived mesenchymal stromal cell spheroids. J Mater Sci Mater Med..

[39] Lee JH, Han Y-S, Lee SH (2016). Long-duration three-dimensional spheroid culture promotes angiogenic activities of adipose-derived mesenchymal stem cells. Biomol Ther (Seoul).

[40] Wang C-C, Chen C-H, Hwang S-M, Lin W-W, Huang C-H, Lee W-Y (2009). Spherically symmetric mesenchymal stromal cell bodies inherent with endogenous extracellular matrices for cellular cardiomyoplasty. Stem Cells..

[41] Emmert MY, Wolint P, Wickboldt N, Gemayel G, Weber B, Brokopp CE (2013). Human stem cell-based three-dimensional microtissues for advanced cardiac cell therapies. Biomaterials..

[42] Liu B-H, Yeh H-Y, Lin Y-C, Wang M-H, Chen DC, Lee B-H, Hsu S-H (2013). Spheroid formation and enhanced cardiomyogenic potential of adipose-derived stem cells grown on chitosan. Biores Open Access..

[43] Amos PJ, Kapur SK, Stapor PC, Shang H, Bekiranov S, Khurgel M (2010). Human adipose-derived stromal cells accelerate diabetic wound healing: impact of cell formulation and delivery. Tissue Eng Part A..

[44] Li Y, Guo G, Li L, Chen F, Bao J, Shi YJ, Bu H (2015). Three-dimensional spheroid culture of human umbilical cord mesenchymal stem cells promotes cell yield and stemness maintenance. Cell Tissue Res..

[45] Talaei-Khozani T, Borhani-Haghighi M, Ayatollahi M, Vojdani Z (2015). An in vitro model for hepatocyte-like cell differentiation from Wharton’s jelly derived-mesenchymal stem cells by cell-base aggregates. Gastroenterol Hepatol Bed Bench..

[46] Xu Y, Shi T, Xu A, Zhang L (2016). 3D spheroid culture enhances survival and therapeutic capacities of MSCs injected into ischemic kidney. J Cell Mol Med..

[47] Sharma RR, Pollock K, Hubel A, McKenna D (2014). Mesenchymal stem or stromal cells: a review of clinical applications and manufacturing practices. Transfusion..

[48] Burnouf T, Strunk D, Koh MBC, Schallmoser K (2015). Human platelet lysate: replacing fetal bovine serum as a gold standard for human cell propagation?. Biomaterials..

[49] Zheng X, Baker H, Hancock WS, Fawaz F, McCaman M, Pungor E (2006). Proteomic analysis for the assessment of different lots of fetal bovine serum as a raw material for cell culture. Part IV. Application of proteomics to the manufacture of biological drugs. Biotechnol Prog.

[50] Bartosh TJ, Ylostalo JH (2014). Preparation of anti-inflammatory mesenchymal stem/precursor cells (MSCs) through sphere formation using hanging-drop culture technique. Curr Protoc Stem Cell Biol..

[51] Seda Tiğli R, Karakeçili A, Gümüşderelioğlu M (2007). In vitro characterization of chitosan scaffolds: influence of composition and deacetylation degree. J Mater Sci Mater Med..

[52] Yeh H, Liu B, Hsu S (2012). The calcium-dependent regulation of spheroid formation and cardiomyogenic differentiation for MSCs on chitosan membranes. Biomaterials..

[53] Hsu S, Huang G (2013). Substrate-dependent Wnt signaling in MSC differentiation within biomaterial-derived 3D spheroids. Biomaterials..

[54] Messina A, Morelli S, Forgacs G, Barbieri G, Drioli E, De Bartolo L (2015). Self-assembly of tissue spheroids on polymeric membranes. J Tissue Eng Regen Med.

[55] Wang W, Itaka K, Ohba S, Nishiyama N, Chung U, Yamasaki Y, Kataoka K (2009). 3D spheroid culture system on micropatterned substrates for improved differentiation efficiency of multipotent mesenchymal stem cells. Biomaterials..

[56] Zhang K, Yan S, Li G, Cui L, Yin J (2015). In-situ birth of MSCs multicellular spheroids in poly(L-glutamic acid)/chitosan scaffold for hyaline-like cartilage regeneration. Biomaterials..

[57] Lewis EEL, Wheadon H, Lewis N, Yang J, Mullin M, Hursthouse A (2016). A quiescent, regeneration-responsive tissue engineered mesenchymal stem cell bone marrow niche model via magnetic levitation. ACS Nano..

[58] Neto AI, Correia CR, Oliveira MB, Rial-Hermida MI, Alvarez-Lorenzo C, Reis RL, Mano JF (2015). A novel hanging spherical drop system for the generation of cellular spheroids and high throughput combinatorial drug screening. Biomater Sci..

[59] Tung Y-C, Hsiao AY, Allen SG, Torisawa Y, Ho M, Takayama S (2011). High-throughput 3D spheroid culture and drug testing using a 384 hanging drop array. Analyst..

[60] Zhao Z, Gu J, Zhao Y, Guan Y, Zhu XX, Zhang Y (2014). Hydrogel thin film with swelling-induced wrinkling patterns for high-throughput generation of multicellular spheroids. Biomacromolecules..

